# Weapons Evolve Faster Than Sperm in Bovids and Cervids

**DOI:** 10.3390/cells10051062

**Published:** 2021-04-29

**Authors:** Charel Reuland, Leigh W. Simmons, Stefan Lüpold, John L. Fitzpatrick

**Affiliations:** 1 Department of Zoology, Stockholm University, Svante Arrhenius väg 18b, 106 91 Stockholm, Sweden; charel.reuland@zoologi.su.se; 2 Centre for Evolutionary Biology, School of Biological Sciences, The University of Western Australia, Crawley, WA 6009, Australia; leigh.simmons@uwa.edu.au; 3 Department of Evolutionary Biology and Environmental Studies, University of Zurich, Winterthurerstrasse 190, 8057 Zurich, Switzerland; stefan.luepold@ieu.uzh.ch

**Keywords:** male–male contest competition, sperm competition, evolutionary rates analysis, male weaponry, sexual selection, sperm morphology

## Abstract

In polyandrous species, males face reproductive competition both before and after mating. Sexual selection thus shapes the evolution of both pre- and postcopulatory traits, creating competing demands on resource allocation to different reproductive episodes. Traits subject to strong selection exhibit accelerated rates of phenotypic divergence, and examining evolutionary rates may inform us about the relative importance and potential fitness consequences of investing in traits under either pre- or postcopulatory sexual selection. Here, we used a comparative approach to assess evolutionary rates of key competitive traits in two artiodactyl families, bovids (family Bovidae) and cervids (family Cervidae), where male–male competition can occur before and after mating. We quantified and compared evolutionary rates of male weaponry (horns and antlers), body size/mass, testes mass, and sperm morphometrics. We found that weapons evolve faster than sperm dimensions. In contrast, testes and body mass evolve at similar rates. These results suggest strong, but differential, selection on both pre- and postcopulatory traits in bovids and cervids. Furthermore, we documented distinct evolutionary rates among different sperm components, with sperm head and midpiece evolving faster than the flagellum. Finally, we demonstrate that, despite considerable differences in weapon development between bovids and cervids, the overall evolutionary patterns between these families were broadly consistent.

## 1. Introduction

The fundamental difference in gamete size between males and females (i.e., anisogamy) sets the stage for the evolution of Darwinian sex roles, resulting in males being more likely than females to compete for reproductive opportunities [1,2,3]. Sexual selection is therefore expected to favour traits in males that increase their competitive abilities [4]. Before mating (i.e., precopulatory), selection acts on a variety of traits to increase males’ competitive ability, such as increased body size, heightened aggression, or the development of dedicated weaponry [4,5]. However, in species where females mate with multiple males, male–male competition can continue after mating (i.e., postcopulatory) in the form of sperm competition [6,7,8,9]. Perhaps not surprisingly then, sexually selected traits associated with male–male competition, including sexual weapons and sperm number or morphology, are among the most diverse traits observed among animals and frequently exhibit accelerated rates of phenotypic evolution compared to other morphological traits [10,11,12]. However, both sexual weapons and ejaculates can be costly to produce and maintain, e.g., [13,14,15], and theoretical work suggests that male expenditure on traits important during precopulatory episodes of selection should interact with, and potentially influence investment in, traits that are important in postcopulatory episodes of selection [16]. Empirical patterns, however, are ambiguous, with positive, negative and no relationship between pre- and postcopulatory traits being observed across a range of taxa [17,18]. Therefore, it remains unclear whether and how the strength of selection varies among traits important in male–male competition before and after mating.

Direct comparisons of the rates of evolutionary divergence among traits offer a powerful tool for identifying which traits are subject to stronger (or weaker) selective pressures. In onthophagine dung beetles, for example, Simmons and Fitzpatrick [11] inferred patterns of selection on a range of traits important in pre- or postcopulatory sexual selection, respectively, and reported that sexual weapons (i.e., horns) evolved faster than body size, and that body size evolved faster than either sperm length or testes size. In pinnipeds (seals, sea lions and walruses), the evolutionary rate of sexual size dimorphism was seven times higher in haremic than non-haremic species, but similar between these groups for testes mass [10]. Together, these findings highlight that sexual selection can drive rapid evolutionary responses in traits crucial during precopulatory male–male competition, including horns in dung beetles and body size in pinnipeds. Moreover, recent work has demonstrated rapid evolutionary divergence in male sexual ornaments in response to precopulatory sexual selection [19,20,21].

Sexual traits can also evolve rapidly in response to the postcopulatory selective forces, with sperm competition thought to play a particularly important role in driving trait diversification. An increase in sperm number at high levels of sperm competition is arguably one of the most robust responses to sexual selection observed in animals [22]. Moreover, sperm are often described as the most diverse and rapidly evolving cell types [23,24]. In other words, both sperm number and morphology are expected to evolve rapidly. Investing in more sperm-producing tissue to generate greater sperm quantities may provide males with a fertilization advantage by sheer numbers in a raffle [7,25,26]. Conversely, increases in total sperm length or the dimensions of functional sperm components may provide a competitive advantage by increasing sperm swimming speed, sperm longevity, or the ability to displace rival sperm already residing within female storage organs (reviewed in [9,24]). Recent work on both *Drosophila* and Lepidopteran species with heteromorphic sperm showed that the morphology of the fertilizing, nucleus-carrying sperm evolved faster than that of the non-fertilizing, anucleate ones [12].

However, there are three important caveats to keep in mind when assessing evolutionary rates of pre- and postcopulatory traits. First, traits may covary positively or negatively (or not at all) across species, making it essential to consider multiple traits simultaneously when examining their evolutionary divergence [9,27,28,29,30]. Second, traits can be made up of distinct, but functionally integrated parts that may respond differently to selection and/or be constrained by genetic correlations [12,31,32]. Thus, traits may evolve at different rates due to differences in the strength or form of selection acting on them or due to constraints or (genetic) trade-offs among them. Distinguishing between these alternatives can be challenging. For example, sperm cells are composed of the head, midpiece and flagellum, which can evolve at different rates (e.g., in birds [33,34]; *Drosophila* species of the *obscura* group [12]; lizards and snakes [35,36]). Finally, comparisons of rates of evolutionary divergence among traits are available for relatively few taxa. Thus, an open question is how these different patterns of trait evolution can be explained, and a wider taxonomic scope is required.

Bovids (family Bovidae) and cervids (family Cervidae), both diverse groups of large herbivores from the order Artiodactyla and longstanding models for studying sexual selection, represent a well-suited system to compare the evolutionary rates of pre- and postmating sexual traits. Darwin [37] noted the sexually dimorphic expression of horns and antlers in bovids and cervids, respectively, as prime examples of sexually selected weapons used in male–male competition over mating opportunities. Subsequent studies have confirmed the link between higher reproductive success for males that invest in larger weaponry [38,39,40]. Males can invest considerable amounts of energy in growing and maintaining these sexual weapons. For example, horns account for up to 15% of body mass in male bighorn sheep (*Ovis canadensis*) [41], and moose (*Alces alces*) increase their energy requirements up to 20% while developing their antlers [42]. Furthermore, the intensity of intrasexual selection predicts weapon size across both bovids [43] and cervids [44]. Precopulatory competitive traits such as these cranial protrusions and body size play a dual role in ungulate contests, being used to convey individual quality in displays and as competitive tools in direct physical confrontations (reviewed in [45]). Given the allometric relationship between weapon and body size, the relative role of these traits in securing competitive success has been difficult to separate, although rare cases of individuals suffering a break in their weapon but still retaining their relative success in competitive bouts highlight the importance of body size in successful contest outcomes [46]. Consequently, many bovids and cervids show male-biased sexual size dimorphism [47,48], and female defence (which facilitates male attempts to monopolize access to females) is present in about half of all species examined [43,49].

Bovids and cervids also exhibit varying levels of sperm competition. In territorial species, for example, males cannot prevent females from moving between territories and remating [43,50]. Even in species with female-defence polygyny (e.g., red deer, *Cervus elaphus*), females may change harems and copulate with several males during a reproductive cycle [40]. In some ungulate groups, testes mass is positively correlated with levels of sperm competition [51], suggesting that investment in testes, and by proxy sperm number, may lead to fertilization benefits during sperm competition. Sperm morphology has also been linked to fertilization success in these species. In red deer, sperm with a longer flagellum and/or a shorter midpiece swim faster [52,53], and ejaculates with a higher proportion of morphologically normal sperm have higher motility [54]. Such patterns, however, may not be universal or apply to interspecific comparisons, as artiodactyls generally show no association between the levels of sperm competition and either total sperm length or the size of any particular sperm component [55], despite positive associations of sperm length with both sperm swimming speeds and levels of sperm competition across mammals [56,57,58]. Yet, despite ample evidence for sexual selection on male sexual weapons and ejaculate traits in bovids and cervids, there is little indication for direct co-evolution between pre- and postcopulatory sexual traits [49,59].

Here, we compared the rates of phenotypic divergence in pre- and postcopulatory traits among bovids and cervids. We focus on traits primarily linked with male–male competition rather than female choice, as the former represents a powerful form of sexual selection in bovids and cervids. We extend the traditional approach of examining phylogenetic correlations among traits by employing a methodology that compares evolutionary rates among multiple traits on a phylogeny. In so doing, we hope to gain new insight into the evolution of male sexual traits in bovids and cervids. For sperm cells specifically, we further examined the evolution of sperm head, midpiece and flagellum length to contrast evolutionary rates among the individual morphological components.

## 2. Materials and Methods

### 2.1. Data Collection

Length and mass data for a range of sexual and somatic traits of ungulates were compiled from the literature (see Dryad link below for raw data). Male sexual weapon length (*n* = 135;, i.e., antlers in cervids and horns in bovids), sperm head, midpiece and total flagellum length (all *n* = 53), and male muzzle width (*n* = 88) were represented by linear measurements. Although antlers, and to a lesser degree horns, can exhibit complex geometries (e.g., [44]), our analyses focused on sexual weapon length, measured as the curvilinear distance along the main axis from the base to the most distal tip of the weapon. Importantly, in ungulates, weapon length is commonly used as a proxy measure of weapon size (e.g., [60]), consistently predicts the overall strength of precopulatory sexual selection [43,44], and alternative measures of weapon size and complexity are strongly correlated (e.g., [40]). Moreover, weapon length measurements were available for a wider range of species than weapon complexity scores or weapon mass values. Antler and horn length can vary based on male age and size [61]. However, rather than using allometric slopes of the relationship between sexual weapons and body size in our analyses (e.g., [62]), we used mean horn and antler lengths previously compiled in the literature. Following Bro-Jørgensen [43], we argue that mean horn and antler length measures offer a conservative estimate of the strength of sexual selection, which is likely skewed in favour of males with above-average sexual weapon lengths. To study the evolution of sperm, we included sperm head, midpiece and flagellum lengths in our models rather than total sperm length as done in previous studies [11], because sperm components themselves may exhibit different rates of phenotypic diversification [12,33,34], thus revealing more detailed information than total sperm length alone. Finally, we also included male muzzle width, a trait less likely to evolve under sexual selection, as a point of reference in our analyses on linear trait measures. Previous studies with a similar approach have assessed proxy measures of body size (e.g., body width or length; [11,35] or locomotive traits related to body size (e.g., wing length in *Drosophila*, [12]). Similarly, male muzzle width is correlated with body mass in the bovids and cervids considered in our dataset (phylogenetic generalized least squares model, PGLS, with muzzle-width and body mass: λ = 0.83, t_81_ = 19.55, *p* < 0.01; note only *n* = 81 species where data was available on both muzzle width and body mass were present in the phylogeny, also see [63]).

Traits measured using mass values included combined testes mass (*n* = 71) and male body mass (*n* = 135, although note only *n* = 62 species where data was available on both testes and body mass were present in the phylogeny). Larger testes are generally capable of producing more sperm [64,65,66,67], and relative testes size generally increases in response to sperm competition [22]. Thus, we included combined testes mass as a proxy for investment in sperm production. Male body mass, which is positively correlated with combined testes mass (PGLS with testes mass and body mass: λ = 0.40, t_62_ = 5.83, *p* < 0.001), was included as a point of comparison for testes mass evolution.

### 2.2. Phylogenetic Analyses

To account for the relatedness among species in all analyses, we used a time-calibrated molecular phylogeny of Cetartiodactyla [68] and pruned it to the species with complete data for each analysis. Our model of length data included 38 species, while the corresponding model of mass data was based on 60 species (phylogenies for both analyses are presented in Supplementary Figure S1). We performed a further set of analyses focusing on the two major ungulate families: the cervids (*n* = 13 and *n* = 17 for the length and mass models, respectively) and bovids (*n* = 25 and *n* = 43 for the length and mass models, respectively). We conducted these separate models because the weapons differ both structurally and developmentally between these taxa, with cervid antlers growing seasonally while bovid horns are maintained [69]. Thus, the energy requirements of armaments, and thus their relationship with other traits, may differ between these two groups. All statistical analyses were conducted in R version 4.0.0 [70].

### 2.3. Comparing Evolutionary Models

A key assumption in the models used to compare phenotypic divergence among traits (see below) is that trait evolution follows a Brownian motion process, i.e., closely related species tend to exhibit more similar trait values than more distant species [71]. We therefore compared alternative evolutionary models to assess which best described the evolution of each trait in ungulates. Specifically, using the *fitContinuous* function included in the *geiger* package [72], we compared the fit of the Brownian motion model to that of an Ornstein –Uhlenbeck (i.e., traits are pulled towards an evolutionary optimum) and an early burst model of trait evolution (i.e., trait changes occur early in the phylogeny) [73,74].

When assessed across all bovid and cervid species with data available, the Brownian motion model was either preferred or statistically indistinguishable from other models (based on sample size-corrected AICc comparisons of evolutionary models, ΔAICc ≤ 4) for several of the length and mass traits, including weapon length, muzzle width and body mass (Supplementary Table S1). However, when initially assessing sperm head, midpiece, and flagellum length or combined testes mass, the Ornstein-Uhlenbeck model was preferred (ΔAICc > 4 when compared to the alternative Brownian motion model, see Supplementary Table S1). Closer inspection of the distribution of sperm and testes trait values among species, using the *plotTree.wBars* function in the R package *phytools* [75], revealed two cases of considerable differences between the most recently diverged sister species in the phylogeny. Specifically, marked differences existed between *Connochaetes taurinus* and *C. gnou* for sperm length and between *Gazella cuvieri* and *G. leptoceros* for testes mass. To assess if these differences drove the support for an Ornstein-Uhlenbeck model of evolution, we removed these four species from the dataset and repeated all model comparisons. Indeed, the Brownian motion model was now either the preferred evolutionary model or non-statistically different from other models for all sperm traits and testes mass (Supplementary Table S2). Since this reduced dataset satisfied the requirement for a Brownian motion process in Adams’ [71] evolutionary rates comparison (see below), we used this reduced dataset going forward. However, for transparency, we also ran all models with these four species included and found broadly similar results (see Supplementary Table S3).

We also compared evolutionary models within each of the two ungulate families separately. Using the reduced dataset described above, all length and mass traits were again best characterized by a Brownian motion model, or the Brownian motion model was statistically undistinguishable from other models (Supplementary Table S3).

### 2.4. Comparing Evolutionary Rates

To compare rates of evolution among traits, we applied a likelihood approach developed by Adams [71]. This method describes the rate at which traits evolve along a phylogeny by quantifying the observed Brownian rate parameter (σ^2^_obs_). The σ^2^_obs_ for each analysis contrasts the trait value in each species against the phylogenetic mean while incorporating the expected amount of trait change under a BM model of evolution to generate estimates of observed rates of evolution for each trait. When assessing multiple traits simultaneously, the σ^2^_obs_ values generate an evolutionary rate matrix, consisting of the observed evolutionary rates on the diagonal elements and the evolutionary covariation between traits (i.e., the evolutionary correlations) on the off-diagonal elements [71]. Likelihood ratio tests are then used to compare this observed rate model against a model in which all traits are constrained to a common evolutionary rate (σ^2^_common_), which represents a constrained model where all diagonal elements of the rate matrix are the same. The σ^2^_common_ model rate estimates are determined by obtaining the diagonal elements where the rate matrix has the lowest joint likelihood value for all traits [71]. To create unit-less variables and avoid differences in trait scales, which could impact estimates of trait variances, all data were log_10_-transformed prior to analysis [66]. Furthermore, since comparisons between length and mass measurements are problematic given their variances are expected to differ [76], we analysed length and mass measures separately (see [11] for a similar approach). To visualize the error around evolutionary rate parameters, the 95% confidence intervals (CIs) were calculated from the standard errors of the Hessian matrix for the evolutionary rate for each trait [71].

Phenotypic traits frequently covary in their observed evolutionary rate matrix [71]. Indeed, covariation (either positive or negative) in the allocation of resources between pre- and postcopulatory traits is a general theoretical prediction [16]. However, models assuming trait covariation frequently failed to converge. Among ungulates, the correlation between many of the pre- and postcopulatory traits examined in our analyses (i.e., weapons, sperm and testes) is weak or absent [49]. Therefore, the failure of models to converge under the assumption of trait covariance may reflect this weak underlying covariance. Thus, all models assumed no trait covariation, which facilitated model convergence (although we note that the few models that did converge under the assumption of trait covariance yielded comparable results to those presented in the Results). All models converged using a Nelder–Mead optimization parameter. For the length models, which compared more than two traits, we used post hoc pairwise comparisons between all trait combinations to determine which traits exhibited significantly different evolutionary rates. Specifically, post hoc comparisons involved running reduced models to statistically compare the σ^2^_obs_ and σ^2^_common_ between all pairs of traits. Post hoc comparisons were conducted using the same parameters and assumptions as the main models.

## 3. Results

**Figure 1:**
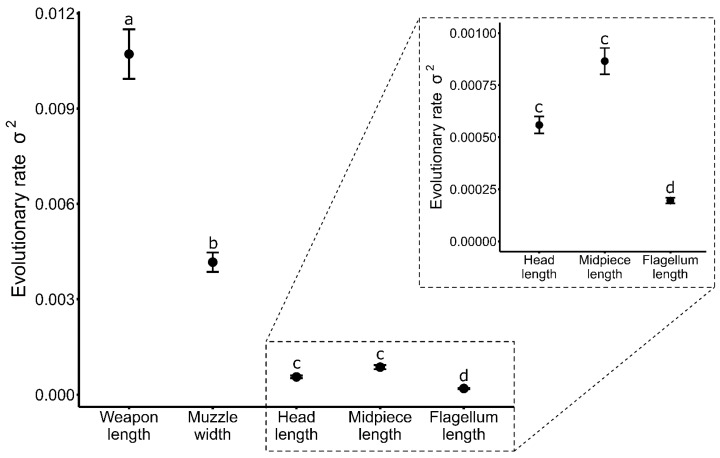
Observed evolutionary rates (σ^2^) of phenotypic divergence and 95% confidence intervals of linear traits (weapon length, muzzle width, sperm head, midpiece and flagellum length) in ungulates. Evolutionary rate estimates for sperm dimensions are magnified in the inset plot. Significant differences between traits are denoted by different letters above each point.

When estimating different evolutionary rates for weapon length, muzzle width and sperm dimensions across all ungulates examined, comparisons of AIC scores indicated that the observed rates of phenotypic divergence differed significantly from the constrained model that assumes a common rate for all length traits (Table 1a). Post hoc pairwise comparisons revealed that most length traits evolved at distinct rates, except for those of sperm head and midpiece lengths that did not differ significantly (Table 1, Figure 1). Weapon length showed the highest rate of phenotypic divergence, evolving approximately 2.6 times faster than muzzle width and 12.5 (sperm head length) to 55 (sperm flagellum length) times faster than any sperm component. Sperm morphology metrics showed the lowest rates of phenotypic divergence, but differed among individual sperm components, with sperm head length evolving approximately 3 times and sperm midpiece length approximately 4.4 times faster than sperm flagellum length (Table 1, see magnified inset plot in Figure 1).

The general differences in evolutionary rates among length measures across all ungulates examined was largely mirrored within families. Weapon length, muzzle width and sperm component lengths evolved at distinct rates in both cervids (LRT = 90.05, *p* < 0.001) and bovids (LRT = 87.07, *p* < 0.001; Supplementary Table S4). Post hoc pairwise comparisons in both families revealed similar patterns of rate differences among traits, except for muzzle width in bovids, which evolved at a rate comparable to weapon length (Supplementary Table S4).

When assessing mass measurements across all ungulates, testes mass and male body mass evolved at a common evolutionary rate (Table 1b), and the same was true within cervids (LRT = 0.25, *p* = 0.62) and bovids (LRT = 2.53, *p* = 0.11, Supplementary Table S4).

## 4. Discussion

Bovids and cervids provide an ideal opportunity to study the coevolution of pre- and postcopulatory male sexual traits, but the evolutionary relationships between these traits have remained elusive [49,77]. In our study, we quantified the evolutionary rates of several traits that influence fitness during either pre- or postcopulatory episodes of sexual selection in ungulates. We found that the length of weapons (i.e., horns or antlers) showed a faster rate of phenotypic evolution than that of sperm head, midpiece and flagellum. Traits subject to strong directional selection, such as sexually selected traits, typically exhibit higher rates of phenotypic diversification [19,78,79,80,81]. Therefore, our findings suggest stronger selective pressures on weaponry than on sperm morphology in ungulates, while testes and body mass evolved at comparable rates. We found no major differences between bovids and cervids, highlighting similar selective pressures acting on trait evolution in both families. Lastly, we uncovered that sperm head and midpiece lengths evolve faster than flagellum length, indicating independent evolution for sperm components. Our analyses therefore illustrate both broad differences in the rate of phenotypic divergence among traits that operate during different episodes of sexual selection and finer differences in the evolutionary rates among sperm components.

Weapon radiations occurred independently in several groups of even-toed ungulates, including cervids and bovids [69]. Consistent with this phenomenon, we found that weapon length showed the highest rate of phenotypic diversification, surpassing both muzzle width and sperm morphometry. The extreme variation of armament size across ungulates has been hypothesized to be largely the result of rapid diversification in social mating systems and associated sexual selection pressures, stemming from frequent habitat changes throughout their evolutionary history (reviewed in [69]). Bovids, for example, underwent an initial radiation in the early Miocene, followed by one or more further radiations during changes in climate and vegetation, such as the development of the savanna [82,83]. Transitions of ungulates from densely forested to more open habitats like grasslands were accompanied by increased group sizes and opportunities for males to monopolize females, which enhanced sexual selection pressures on males and favoured the evolution of larger and more complex weaponry [69]. Transitions between habitat types also changed male fighting styles, thereby further promoting the diversification in male body size and weapon size and complexity [69,84,85,86]. These evolutionary trajectories might ultimately also explain the higher rates of phenotypic divergence of male weapons compared to other sexual and non-sexual traits examined in our study.

We documented slow rates of evolution for sperm morphological traits, which appears at odds with the large diversity in sperm morphology commonly observed among animals [23,24]. Our finding of low rates of phenotypic diversity of sperm components, however, is in line with previous research on sperm evolution. In onthopagine dung beetles, sperm length exhibited slower rates of evolution than male body size and horn length [11]. Similarly, testis size as well as male body length of anole lizards have evolved faster than any aspect of sperm morphology [35]. Low rates of phenotypic diversification may occur if postcopulatory sexual selection on sperm morphology is stabilizing rather than directional. Indeed, strong postcopulatory selection is hypothesized, and has been demonstrated, to reduce variance in sperm traits both within species and ejaculates [25,87,88,89,90,91,92,93]. However, it is currently unclear if such selective pressures are also applying to interspecific diversification, for example in ungulates. Alternatively, low rates of evolution in sperm size could emerge if an evolutionary trade-off exists between sperm size and number and sperm number is the primary target of selection, relaxing selection on sperm length. Indeed, in larger-bodied mammals like ungulates, the combined selection through sperm competition and the risk of sperm dilution within the female reproductive tract favours sperm number over size [27]. Alternatively, mass-specific metabolic rate may act as a constraint on sperm size, and large animals like ungulates with low mass-specific metabolic rates may produce comparatively smaller sperm as they are unable to process resources at the rates needed to achieve a large sperm size [55,94]. Finally, sperm morphology may exhibit lower evolutionary rates simply because they are not the primary target of selection in ungulates. Future research on ungulates should therefore focus on differences in sperm morphology and associated fertilization benefits to better understand the evolution of sperm cells.

Although our findings suggest that sperm are not always the most rapidly evolving trait, it should be noted that comparing single-cell gametes with complex, multicellular traits might have its caveats. Sperm themselves are often regarded as the most diverse cell type known [23,24] and may indeed demonstrate faster rates of phenotypic evolution than other cell types. Yet, overall, modifications to a single cell, such as the size of different sperm components, may be more constrained than modifications to large, multicellular structures such as male weaponry, resulting in divergent rates of phenotypic diversification between these traits. Furthermore, unlike the evolution of the shape and size of external weapons, the selective environment within the female reproductive tract might impose strong constraints on the evolution of sperm dimensions. However, contrasting evolutionary constraints can be challenging. Horns and antlers likely also face developmental and physiological constraints (e.g., [95]), and determining how these constraints compare to those experienced by sperm and subsequently influence their evolutionary trajectories is not straightforward. An interesting future avenue for research would thus be to compare evolutionary rates among traits that share similar potential for evolutionary constraints (e.g., comparing sperm morphology to other single-cell structures, or between different ejaculate traits).

Our analysis revealed that both sperm head and midpiece length evolved faster than flagellum length. Sperm components may evolve independently and exhibit alternative patterns of genetic covariance [31,96]. In passerine birds, for example, the midpiece and flagellum exhibit a concerted response to sexual selection, whereas sperm head length appears to be evolutionarily constrained [33,34], potentially owing to functional constraints due to genome size [97,98,99]. In contrast, midpiece length evolved at a faster rate than sperm head and flagellum length in anole lizards [35]. Further, heteromorphic sperm of the *Drosophila obscura* species group show a complex evolutionary pattern, with head length evolving twice as fast as flagellum length in fertilizing sperm but over four times more slowly in non-fertilizing sperm [12]. Our results suggest that in ungulates, the strength of selection may differ between sperm flagellum length and other sperm components, with the flagellum being either under relaxed selection, stabilizing selection, or constrained in its response to selection (e.g., due to trade-offs between sperm size and number). Conversely, the sperm head and midpiece exhibit a more concerted evolutionary trajectory. It thus seems that sperm components show different patterns of evolution between all four taxa studied so far, with ungulates exhibiting another previously undocumented pattern of sperm evolution.

Larger body size is linked to increased success at displacing rival males, and many bovid and cervid species thus exhibit male-biased sexual size dimorphism [47,48]. In our analyses, the evolutionary rates of body mass were on par with testes mass, suggesting that the latter may play an equally crucial role in attaining a competitive advantage. It thus seems that postcopulatory sexual selection may primarily target testes mass, and by inference sperm number, rather than sperm morphology. Such an effect is supported by previous studies demonstrating that testes mass, but not sperm component or total length, is positively correlated with the level of sperm competition across ungulates [51,55]. Furthermore, some ungulate species are known to face sperm depletion during the mating season, suggesting there can be intense selection on sperm production in this group [100,101]. Our findings for ungulates, however, contrast with those reported for onthophagine dung beetles, where testes mass showed a reduced rate of phenotypic divergence compared to body mass [11]. Sperm dilution effects may result in stronger selection for sperm number and thus testes mass in larger-bodied species such as ungulates (e.g., [27]) compared to small-bodied species like dung beetles where females store sperm in specially adapted sperm storage organs [11], potentially explaining the different patterns found here. Alternatively, these distinct evolutionary trajectories could result from different absolute and relative investments in male weapons and testes between these taxa and the role of precopulatory contests vs. sperm competition in determining reproductive outcomes (e.g., see [59]).

In conclusion, our findings suggest accelerated rates of phenotypic divergence in weapons compared to sperm morphology, but similar selective pressures on body and testes mass in even-toed ungulates. Within sperm cell morphometry, we found greater rates of evolutionary divergence in sperm head and midpiece lengths in comparison to flagellum length, a unique pattern that warrants further studies to elucidate the evolutionary causes and consequences.

## Figures and Tables

**Table 1:** Comparisons of evolutionary rates of (a) length measures and (b) mass measures in ungulates. The model (a) compares length measures (i.e., horn/antler length, muzzle width and sperm head, midpiece and flagellum length), while model (b) compares mass measures (testes and body mass). Note that the two models assess different numbers of ungulate species. The observed (σ^2^_obs_) and common evolutionary rate (σ^2^_common_) are shown for each trait. Additionally, presented are the AIC values for the observed (AIC_obs_) and common (AIC_common_) model, log-likelihood values for the observed (Log(L_obs_)) and common models (Log(L_common_)), and the log-likelihood ratio tests (LRT) and associated p-values for comparisons between observed and constrained rate models. Log-likelihood values, LRTs, and p-values are further displayed for Post hoc pairwise comparisons.

Trait	σ^2^_obs_	σ^2^_common_	AIC_obs_	AIC_common_	Log (L_obs_)	Log (L_common_)	LRT	*p*
(a) Length Measure Comparisons (*n* = 38)								
Horn/antler length	10.71 × 10^−3^	3.30 × 10^−3^	−245.97	−81.83	132.98	46.91	172.14	<0.001
Muzzle width	4.17 × 10^−3^							
Sperm head length	0.56 × 10^−3^							
Sperm midpiece length	0.87 × 10^−3^							
Sperm flagellum length	0.20 × 10^−3^							
Post hoc pairwise comparisons	Head length vs. Midpiece length				77.96	77.05	1.81	0.18
	Head length vs. Flagellum length				106.20	101.20	1.00	<0.01
	Head length vs. Muzzle width				48.09	31.47	33.25	<0.001
	Head length vs. Weapon length				30.15	−1.58	63.45	<0.001
	Midpiece length vs. Flagellum length				97.88	88.23	19.30	<0.001
	Midpiece length vs. Muzzle width				39.77	29.08	21.39	<0.001
	Midpiece length vs. Weapon length				21.82	−2.60	48.85	<0.001
	Flagellum length vs. Muzzle width				68.02	34.51	67.02	<0.001
	Flagellum length vs. Weapon length				50.07	−0.34	100.81	<0.001
	Muzzle width vs. Weapon length				−8.04	−12.13	8.18	<0.01
(b) Mass Measure Comparisons (*n* = 60)								
Testes mass	0.019	0.017	118.56	117.42	−55.28	−55.71	0.87	0.35
Body mass	0.015							

## Data Availability

All data were collected from the literature. Analyses and datasets are available in Dryad (https://doi.org/10.5061/dryad.dbrv15f19, accessed on 23 April 2021).

## Supplementary Materials

The following are available online at https://www.mdpi.com/article/10.3390/cells10051062/s1, Table S1: Comparison of model fit for each trait assuming Brownian motion, Ornstein-Uhlenbeck or early burst evolutionary models—full dataset without removing species to fulfil the assumption of Brownian motion. Table S2: Comparisons of evolutionary rates of (a) length measures and (b) mass measures in ungulates—full dataset without cut species to fulfil assumption of Brownian motion. Table S3: Comparison of model fit for each trait assuming Brownian motion, Ornstein-Uhlenbeck or Early burst evolutionary models. Table S4: Comparisons of evolutionary rates of length measures in cervids (a) and bovids (b), as well as mass measures in cervids (c) and bovids (d).
