# Supplementary material for: Weapons Evolve Faster Than Sperm in Bovids and Cervids
Source: Cells. 2021 Apr 29;10(5):1062. doi: 10.3390/cells10051062 (PMC8145498; doi:10.3390/cells10051062)
Supplement: Supplementary file 1 [file cells-10-01062-s001.zip › cells-1176477-supplementary.pdf]

cells MDPI

# Weapons Evolve Faster Than Sperm in Bovids and Cervids

**Charel Reuland [1], Leigh W. Simmons [2], Stefan Lüpold [3,†] and John L. Fitzpatrick [1,†,*]**

1 Department of Zoology, Stockholm University, Svante Arrhenius väg 18b, 106 91 Stockholm, Sweden; charel.reuland@zoologi.su.se
2 Centre for Evolutionary Biology, School of Animal Biology (M092), The University of Western Australia, Crawley, WA 6009, Australia; leigh.simmons@uwa.edu.au
3 Department of Evolutionary Biology and Environmental Studies, University of Zurich, Winterthurerstrasse 190, 8057 Zurich, Switzerland; stefan.luepold@ieu.uzh.ch
* Correspondence: john.fitzpatrick@zoologi.su.se
† Equal contribution

## Supplementary Figures and Tables

## Supplementary Figure S1

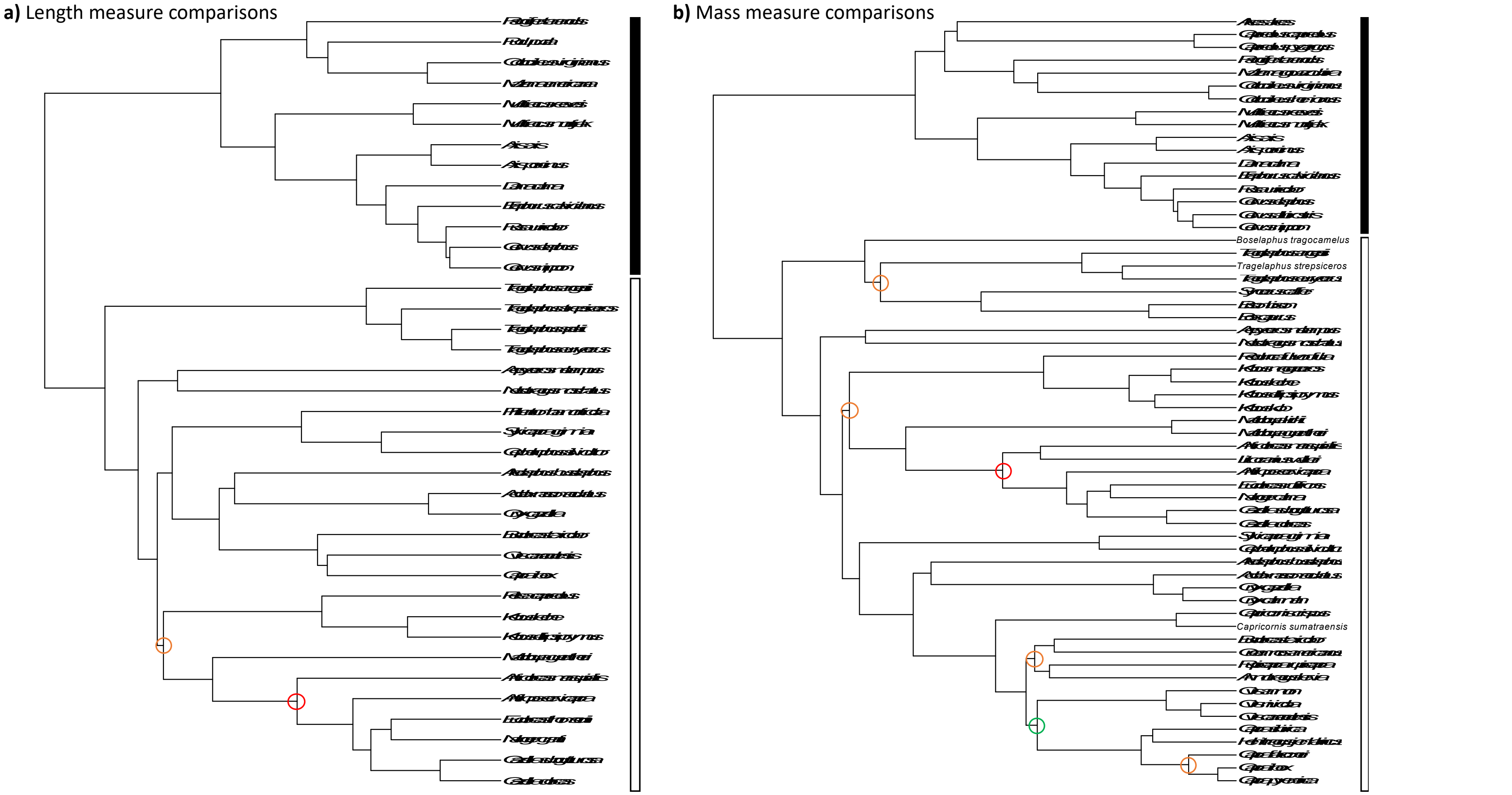


**Supplementary Figure S1. Comparing evolutionary rates in bovids and cervids.** Phylogenetic relationships of bovid and cervid species examiend in **a)** length measure comparisions (n = 38), which contrasted evolutionary rates of weapon length, muzzel width and sperm head, midpeice and flagellum length), and **b)** mass measure comparisions (n = 60), which compared testes and body mass. The white and black bars on the right side of each plot indicates bovids (family Bovidae) and cervids (family Cervidae), respectivley. The phylogenies were pruned from Zurano et al.'s (2019) time-calibrated molecular phylogeny of Cetartiodactyla. Some of the nodal values in Zurano et al.'s (2019) phylogeny had

relatively low support (i.e. posterior probabilities). Because rates estimates are sensitive to phylogenetic certainty, we labelled all nodal values where Zurano et al. (2019) indicated posterior probabilities are < 0.91 (we maintained Zurano et al.'s colour scheme and use red, orange and green circles to depict nodes with posterior probabilities < 0.60, between 0.61-0.80, and 0.81-0.90, respectively. All unlabelled nodes had posterior probabilities > 0.91. In the species included in our dataset, relatively lower support was only present among bovids. Therefore, our anaylses that assessed bovids and cervids separately allow us to focus one groups where there either is more (bovids) or less (cervids) phylogenetic uncertainy among species.

**Supplementary Table S1: Comparison of model fit for each trait assuming Brownian motion, Ornstein-Uhlenbeck or early-burst evolutionary models - full dataset without removing species to fulfil the assumption of Brownian motion.** Note that these models include four species removed from the main analysis, *Connochaetes taurinus*, *C. gnou*, *Gazella cuvieri*, and *G. leptoceros*, which prevented the models from following a Brownian-motion model (see table). Presented are the Brownian rate parameter $\sigma^2$, the selection strength parameter $\alpha$ and the rate of evolutionary change parameter $a$ for the respective models. Maximum likelihood estimates (lnL) and the sample size corrected Akaike Information Criterion (AICc) are given for each trait. Akaike weights ($\omega_i$) represent the strength of evidence for each model, ΔAICc ($\Delta_i$) denotes the differences between the current and lowest AICc values, with values < 4 indicating no statistically significant difference between models [66]. The best fitting model is indicated in bold text for each trait.

| | Brownian motion model | | | | | Ornstein-Uhlenbeck model | | | | | Early burst model | | | | |
|---|---|---|---|---|---|---|---|---|---|---|---|---|---|---|---|
| **Trait** | $\sigma^2$ | **lnL** | **AICc** | $\Delta_i$ | $\omega_i$ | $\alpha$ | **lnL** | **AICc** | $\Delta_i$ | $\omega_i$ | $a$ | **lnL** | **AICc** | $\Delta_i$ | $\omega_i$ |
| **(a) Length measurements (n = 40)** | | | | | | | | | | | | | | | |
| Head length | $9.78 * 10^{-4}$ | 34.99 | -65.66 | 17.37 | $1.69 * 10^{-4}$ | **22022.55** | **44.84** | **-83.02** | **0.00** | **1.00** | $-1.00 * 10^{-6}$ | 35.00 | -63.31 | 19.71 | $5.35 * 10^{-4}$ |
| Midpiece length | $1.16 * 10^{-3}$ | 31.56 | -58.79 | 6.34 | 0.04 | **0.15** | **35.90** | **-65.13** | **0.00** | **0.95** | $-1.00 * 10^{-6}$ | 31.56 | -56.45 | 8.68 | 0.01 |
| Flagellum length | $2.92 * 10^{-4}$ | 59.19 | -114.06 | 15.33 | $4.68 * 10^{-4}$ | **22024.67** | **68.03** | **-129.39** | **0.00** | **1.00** | $-1.00 * 10^{-6}$ | 59.19 | -111.72 | 17.67 | $1.45 * 10^{-4}$ |
| Muzzle width | $\mathbf{4.04 * 10^{-3}}$ | **6.64** | **-8.96** | **0.00** | **0.61** | $1.63 * 10^{-2}$ | 6.73 | -6.78 | 2.17 | 0.20 | $-1.00 * 10^{-6}$ | 6.64 | -6.61 | 2.34 | 0.19 |
| Weapon length | $\mathbf{1.02 * 10^{-2}}$ | **-11.91** | **28.14** | **0.00** | **0.61** | $1.65 * 10^{-2}$ | -11.82 | 30.31 | 2.17 | 0.21 | $-1.00 * 10^{-6}$ | -11.91 | 30.49 | 2.34 | 0.19 |
| **(b) Mass measurements (n = 64)** | | | | | | | | | | | | | | | |
| Testes mass | $4.06 * 10^{-2}$ | -54.84 | 113.87 | 27.50 | $1.07 * 10^{-6}$ | **0.31** | **-39.97** | **86.37** | **0.00** | **1.00** | $-1.00 * 10^{-6}$ | -54.84 | 116.07 | 29.70 | $3.55 * 10^{-7}$ |
| Body mass | $\mathbf{1.42 * 10^{-2}}$ | **-21.22** | **46.63** | **0.00** | **0.52** | $8.36 * 10^{-17}$ | -21.22 | 48.83 | 2.20 | 0.17 | $-5.13 * 10^{-2}$ | -20.61 | 47.63 | 1.00 | 0.31 |

**Supplementary Table S3: Comparison of model fit for each trait assuming Brownian motion, Ornstein-Uhlenbeck or Early-burst evolutionary models.** Presented are the Brownian rate parameter $\sigma^2$, the selection strength parameter $\alpha$ and the rate of evolutionary change parameter *a* for the respective models. Maximum likelihood estimates (InL) and sample size corrected Akaike Information Criterion (AICc) are given for each trait. Akaike weights ($\omega_i$) represent the strength of evidence for each model, Delta AICc ($\Delta_i$) denotes the differences between the current and lowest AICc values, with values < 4 indicating no statistically difference between models [66]. The best fitting model is indicated in bold text for each trait.

| | Brownian motion model | | | | | Ornstein-Uhlenbeck model | | | | | Early burst model | | | | |
|---|---|---|---|---|---|---|---|---|---|---|---|---|---|---|---|
| **Trait** | **$\sigma^2$** | **lnL** | **AICc** | **$\Delta_i$** | **$\omega_i$** | **$\alpha$** | **lnL** | **AICc** | **$\Delta_i$** | **$\omega_i$** | ***a*** | **lnL** | **AICc** | **$\Delta_i$** | **$\omega_i$** |
| **(a) Ungulate length measurements (n = 38)** | | | | | | | | | | | | | | | |
| Head length | 5.58 * $10^{-4}$ | 43.14 | -81.9 | 2.30 | 0.22 | **0.10** | **45.47** | **-84.24** | **0.00** | **0.71** | -1.00 * $10^{-6}$ | 43.14 | -79.57 | 4.66 | 0.07 |
| Midpiece length | 8.65 * $10^{-4}$ | 34.82 | -65.29 | 1.13 | 0.33 | **8.38 * $10^{-2}$** | **36.57** | **-66.43** | **0.00** | **0.57** | -1.00 * $10^{-6}$ | 34.82 | -62.93 | 3.49 | 0.10 |
| Flagellum length | 1.96 * $10^{-4}$ | 63.06 | -121.78 | 2.83 | 0.18 | **0.16** | **65.66** | **-124.61** | **0.00** | **0.76** | -1.00 * $10^{-6}$ | 63.06 | -119.42 | 5.19 | 0.06 |
| Muzzle width | **4.17 * $10^{-3}$** | **4.96** | **-5.57** | **0.00** | **0.58** | 3.37 * $10^{-2}$ | 5.25 | -3.79 | 1.78 | 0.24 | -1.00 * $10^{-6}$ | 4.96 | -3.21 | 2.36 | 0.18 |
| Weapon length | **1.07 * $10^{-2}$** | **-12.99** | **30.33** | **0.00** | **0.59** | 2.70 * $10^{-2}$ | -12.78 | 32.28 | 1.95 | 0.22 | -1.00 * $10^{-6}$ | -12.99 | 32.69 | 2.36 | 0.18 |
| **(b) Cervid length measurements (n = 13)** | | | | | | | | | | | | | | | |
| Head length | **3.42 * $10^{-4}$** | **19.94** | **-34.69** | **0.00** | **0.66** | 0.13 | 20.60 | -32.53 | 2.16 | 0.22 | -1.00 * $10^{-6}$ | 19.94 | -31.22 | 3.47 | 0.12 |
| Midpiece length | **9.45 * $10^{-4}$** | **13.34** | **-21.49** | **0.00** | **0.71** | 6.53 * $10^{-2}$ | 13.65 | -18.64 | 2.85 | 0.17 | -1.00 * $10^{-6}$ | 13.35 | -18.02 | 3.47 | 0.12 |
| Flagellum length | **7..70 * $10^{-5}$** | **29.64** | **-54.08** | **0.00** | **0.71** | 5.90 * $10^{-2}$ | 29.92 | -51.17 | 2.91 | 0.17 | -1.00 * $10^{-6}$ | 29.64 | -50.62 | 3.47 | 0.13 |
| Muzzle width | **3.90 * $10^{-3}$** | **4.14** | **-3.08** | **0.00** | **0.70** | 0.12 | 4.53 | -0.39 | 2.70 | 0.18 | -1.00 * $10^{-6}$ | 4.14 | 0.38 | 3.47 | 0.12 |
| Weapon length | **1.68 * $10^{-2}$** | **-5.35** | **15.90** | **0.00** | **0.70** | 0.85 | -5.00 | 18.67 | 2.77 | 0.18 | -1.00 * $10^{-6}$ | -5.35 | 19.37 | 3.47 | 0.12 |
| **(c) Bovid length measurements (n = 25)** | | | | | | | | | | | | | | | |
| Head length | 6.69 * $10^{-4}$ | 25.06 | -45.57 | 0.93 | 0.35 | **0.12** | **26.82** | **-46.50** | **0.00** | **0.56** | -1.00 * $10^{-6}$ | 25.06 | -42.98 | 3.53 | 0.10 |
| Midpiece length | **8.21 * $10^{-4}$** | **22.52** | **-40.49** | **0.00** | **0.49** | 9.63 * $10^{-2}$ | 23.54 | -39.94 | 0.55 | 0.37 | -1.00 * $10^{-6}$ | 22.52 | -37.89 | 2.60 | 0.13 |
| Flagellum length | 2.60 * $10^{-4}$ | 37.04 | -69.53 | 3.03 | 0.17 | **0.34** | **39.85** | **-72.56** | **0.00** | **0.78** | -1.00 * $10^{-6}$ | 37.04 | -66.94 | 5.63 | 0.05 |
| Muzzle width | **4.30 * $10^{-3}$** | **1.81** | **0.93** | **0.00** | **0.65** | 8.72* $10^{-3}$ | 1.82 | 3.50 | 2.56 | 0.18 | -1.00 * $10^{-6}$ | 1.81 | 3.53 | 2.60 | 0.18 |
| Weapon length | **7.56 * $10^{-3}$** | **-5.24** | **15.03** | **0.00** | **0.44** | 5.14 * $10^{-18}$ | -5.24 | 17.62 | 2.60 | 0.12 | -0.15 | -3.97 | 15.08 | 0.05 | 0.43 |
| **(e) Ungulate mass measurements (n = 60)** | | | | | | | | | | | | | | | |
| Testes mass | 1.89 * $10^{-2}$ | -31.25 | 66.71 | 3.54 | 0.14 | **6.91 * $10^{-2}$** | **-28.37** | **63.17** | **0.00** | **0.82** | -1.00 * $10^{-6}$ | -31.25 | 68.93 | 5.76 | 0.05 |
| Body mass | **1.48 * $10^{-2}$** | **-24.03** | **52.27** | **0.00** | **0.56** | 1.47 * $10^{-16}$ | -24.03 | 54.49 | 2.22 | 0.18 | -4.01 * $10^{-2}$ | -23.69 | 53.82 | 1.54 | 0.26 |
| **(f) Cervid mass measurements (n = 17)** | | | | | | | | | | | | | | | |
| Testes mass | **1.74 * $10^{-2}$** | **-6.33** | **17.51** | **0.00** | **0.59** | 9.42 * $10^{-2}$ | -5.59 | 19.02 | 1.51 | 0.28 | -1.00 * $10^{-6}$ | -6.33 | 20.50 | 2.99 | 0.13 |
| Body mass | **2.21 * $10^{-2}$** | **-8.38** | **21.61** | **0.00** | **0.67** | 5.45 * $10^{-2}$ | -8.17 | 24.19 | 2.58 | 0.18 | -1.00 * $10^{-6}$ | -8.38 | 24.60 | 2.99 | 0.15 |
| **(g) Bovid mass measurements (n = 43)** | | | | | | | | | | | | | | | |
| Testes mass | 1.95 * $10^{-2}$ | -23.77 | 51.84 | 1.11 | 0.33 | **5.77 * $10^{-2}$** | **-22.06** | **50.73** | **0.00** | **0.57** | -1.00 * $10^{-6}$ | -23.77 | 54.15 | 3.42 | 0.10 |

| | | | | | | | | | | | | | | |
|---|---|---|---|---|---|---|---|---|---|---|---|---|---|---|
| Body mass | $1.20 * 10^{-2}$ | -13.29 | 30.88 | 2.33 | 0.22 | $8.30 * 10^{-18}$ | -13.29 | 33.20 | 4.65 | $6.94 * 10^{-2}$ | **-0.11** | **-10.97** | **28.55** | **0.00** | **0.71** |

**Supplementary Table S3: Comparisons of evolutionary rates of (a) length measures and (b) mass measures in ungulates - full dataset without cut species to fulfil assumption of Brownian motion.** Note that these models include four species removed from the main analysis, *Connochaetes taurinus*, *Connochaetes gnou*, *Gazella cuvieri*, and *Gazella leptoceros*, but that postcopulatory traits no longer follow the assumption of a Brownian motion process of evolution. The model comparing (a) length measures included horn/antler length, muzzle width and sperm head, midpiece and flagellum length, while the model comparing (b) mass measures included testes and body mass. Note that the two models assess different numbers of ungulate species. The observed evolutionary rate ($\sigma^2_{obs}$) and common ($\sigma^2_{common}$) rate are shown for each trait. Also presented are the AIC values for the observed ($AIC_{obs}$) and common ($AIC_{common}$) model, log-likelihood values for the observed ($Log(L_{obs})$) and common models ($Log(L_{common})$), log-likelihood ratio tests (LRT) which compare models of observed rates against a constrained model where all traits evolve at a common rate, and *p*-values. Log-likelihood values and ratio tests, as well as p-value are furthermore displayed for Post hoc pairwise comparisons where two traits where analysed at a time. Significant *p*-values are presented in bold text. Overall, the findings were qualitatively similar for the length model (compare Table 1a and Supplementary Table S2a), but testes and body mass showed significantly different evolutionary rates when all species were included (compare Table 1b and Supplementary Table S2b). However, we urge readers to interpret this significant result with utmost caution, as the underlying assumptions of the methodology about trait evolution were not met for these Supplementary models. We hope that future research can address this interesting finding of postcopulatory trait evolution possibly resembling an Ornstein-Uhlenbeck process in ungulates using statistical approach that is capable of comparing evolutionary rates among multiple traits under an Ornstein-Uhlenbeck model.

## Supplementary Table S3

| Trait | $\sigma^2_{obs}$ | $\sigma^2_{common}$ | $AIC_{obs}$ | $AIC_{common}$ | Log ($L_{obs}$) | Log ($L_{common}$) | LRT | p |
|---|---|---|---|---|---|---|---|---|
| **(a) Length measure comparisons (n = 40)** | | | | | | | | |
| Horn/antler length | $10.2 * 10^{-3}$ | $3.33 * 10^{-3}$ | -220.94 | -92.61 | 120.47 | 52.31 | 136.33 | **<0.001** |
| Muzzle width | $4.04 * 10^{-3}$ | | | | | | | |
| Sperm head length | $0.98 * 10^{-3}$ | | | | | | | |
| Sperm midpiece length | $1.16 * 10^{-3}$ | | | | | | | |
| Sperm flagellum length | $0.29 * 10^{-3}$ | | | | | | | |
| **Post-hoc pairwise comparisons:** | Head length vs. Midpiece length | | | | 66.55 | 66.40 | 0.29 | 0.59 |
| | Head length vs. Flagellum length | | | | 94.18 | 87.27 | 13.83 | **<0.001** |
| | Head length vs. Muzzle width | | | | 41.63 | 32.33 | 18.61 | **<0.001** |
| | Head length vs. Weapon length | | | | 23.08 | 0.244 | 45.67 | **<0.001** |
| | Midpiece length vs. Flagellum length | | | | 90.75 | 81.88 | 17.74 | **<0.001** |
| | Midpiece length vs. Muzzle width | | | | 38.20 | 30.89 | 14.61 | **<0.001** |
| | Midpiece length vs. Weapon length | | | | 19.65 | -0.40 | 40.11 | **<0.001** |
| | Flagellum length vs Muzzle width | | | | 65.83 | 38.22 | 55.23 | **<0.001** |
| | Flagellum length vs. Weapon length | | | | 47.28 | 2.78 | 89.01 | **<0.001** |
| | Muzzle width vs. Weapon length | | | | -5.27 | -9.43 | 8.31 | **<0.01** |
| **(b) Mass measure comparisons (n = 64)** | | | | | | | | |
| Testes mass | 0.041 | 0.027 | 160.11 | 175.01 | -76.05 | -84.51 | 16.91 | **<0.001** |
| Body mass | 0.014 | | | | | | | |

**Supplementary Table S4: Comparisons of evolutionary rates of length measures in cervids (a) and bovids (b), as well as mass measures in cervids (c) and bovids (d).** The models comparing length measures (a + b) included horn/antler length, muzzle width and sperm head, midpiece and flagellum length, while the models comparing mass measures (c + d) included testes and body mass. Note that the four models assess different numbers of cervid and bovid species. The observed evolutionary rate ($\sigma^2_{obs}$) and common ($\sigma^2_{common}$) rate are shown for each trait. Also presented are the AIC values for the observed ($AIC_{obs}$) and common ($AIC_{common}$) model, log-likelihood values for the observed ($Log(L_{obs})$) and common models ($Log(L_{common})$), log-likelihood ratio tests (LRT) which compare models of observed rates against a constrained model where all traits evolve at a common rate, and *p*-values. Log-likelihood values and ratio tests, as well as p-value are furthermore displayed for Post hoc pairwise comparisons where two traits where analysed at a time. Significant *p*-values are presented in bold text.

| Trait | $\sigma^2_{obs}$ | $\sigma^2_{common}$ | $AIC_{obs}$ | $AIC_{common}$ | $Log (L_{obs})$ | $Log (L_{common})$ | LRT | p |
|---|---|---|---|---|---|---|---|---|
| **(a) Cervidae length measure comparisons (n = 13)** | | | | | | | | |
| Horn/antler length | $16.76 * 10^{-3}$ | $4.40 * 10^{-3}$ | -103.44 | -21.39 | 61.72 | 16.70 | 90.05 | **<0.001** |
| Muzzle width | $3.89 * 10^{-3}$ | | | | | | | |
| Sperm head length | $0.34 * 10^{-3}$ | | | | | | | |
| Sperm midpiece length | $0.95 * 10^{-3}$ | | | | | | | |
| Sperm flagellum length | $0.08 * 10^{-3}$ | | | | | | | |
| **Post-hoc pairwise comparisons:** | | Head length vs. Midpiece length | | | 33.29 | 31.68 | 3.21 | *0.07* |
| | | Head length vs. Flagellum length | | | 49.59 | 46.26 | 6.65 | **<0.01** |
| | | Head length vs. Muzzle width | | | 24.08 | 16.20 | 15.77 | **<0.001** |
| | | Head length vs. Weapon length | | | 14.59 | -1.95 | 33.09 | **<0.001** |
| | | Midpiece length vs. Flagellum length | | | 42.99 | 34.68 | 16.61 | **<0.001** |
| | | Midpiece length vs. Muzzle width | | | 17.49 | 14.47 | 6.03 | **<0.05** |
| | | Midpiece length vs. Weapon length | | | 8.00 | -2.40 | 20.79 | **<0.001** |

| | | | | | | | | |
|---|---|---|---|---|---|---|---|---|
| | | Flagellum length vs Muzzle width | | | 33.78 | 17.04 | 33.49 | **<0.001** |
| | | Flagellum length vs. Weapon length | | | 24.29 | -1.75 | 52.08 | **<0.001** |
| | | Muzzle width vs. Weapon length | | | -1.21 | -4.40 | 6.39 | **<0.05** |
| **(b) Bovidae length measure comparisons (n = 25)** | | | | | | | | |
| Horn/antler length | 7.56 * $10^{-3}$ | 2.72 * $10^{-3}$ | -142.37 | -63.29 | -14.70 | -14.83 | 0.25 | 0.62 |
| Muzzle width | 4.30 * $10^{-3}$ | | | | | | | |
| Sperm head length | 0.67 * $10^{-3}$ | | | | | | | |
| Sperm midpiece length | 0.82 * $10^{-3}$ | | | | | | | |
| Sperm flagellum length | 0.26 * $10^{-3}$ | | | | | | | |
| | **Post-hoc pairwise comparisons:** | Head length vs. Midpiece length | | | 47.58 | 47.45 | 0.26 | 0.61 |
| | | Head length vs. Flagellum length | | | 62.10 | 59.33 | 5.53 | **<0.05** |
| | | Head length vs. Muzzle width | | | 26.87 | 17.33 | 19.08 | **<0.001** |
| | | Head length vs. Weapon length | | | 19.82 | 4.73 | 30.19 | **<0.001** |
| | | Midpiece length vs. Flagellum length | | | 59.56 | 55.56 | 8.00 | **<0.01** |
| | | Midpiece length vs. Muzzle width | | | 24.32 | 16.58 | 15.49 | **<0.001** |
| | | Midpiece length vs. Weapon length | | | 17.28 | 4.27 | 26.01 | **<0.001** |
| | | Flagellum length vs Muzzle width | | | 38.85 | 19.49 | 38.71 | **<0.001** |
| | | Flagellum length vs. Weapon length | | | 31.80 | 6.01 | 51.57 | **<0.001** |
| | | Muzzle width vs. Weapon length | | | -3.43 | -4.41 | 1.96 | 0.16 |
| **(c) Cervidae mass measure comparisons (n = 17)** | | | | | | | | |
| Testes mass | 0.017 | 0.020 | 37.41 | 35.65 | -14.70 | -14.83 | 0.25 | 0.62 |
| Body mass | 0.022 | | | | | | | |
| **(d) Bovidae mass measure comparisons (n = 43)** | | | | | | | | |
| Testes mass | 0.020 | 0.016 | 82.12 | 82.65 | -37.06 | -38.32 | 2.53 | 0.11 |

| | |
|---|---|
| Body mass | 0.012 |

## Supplemental References

Zurano, J.P.; Magalhães, F.M.; Asato, A.E.; Silva, G.; Bidau, C.J.; Mesquita, D.O.; Costa, G.C. Cetartiodactyla: updating a time-calibrated molecular phylogeny. *Mol. Phylogenet. Evol.* **2019**, *133*, 256–262, doi:10.1016/j.ympev.2018.12.015.
